# Genes co-amplified with *ERBB2* or *MET* as novel potential cancer-promoting genes in gastric cancer

**DOI:** 10.18632/oncotarget.21150

**Published:** 2017-09-21

**Authors:** Mi Jeong Kwon, Ryong Nam Kim, Kyoung Song, Sinyoung Jeon, Hae Min Jeong, Joo Seok Kim, Jinil Han, Sungyoul Hong, Ensel Oh, Jong-Sun Choi, Jungsuk An, Jonathan R. Pollack, Yoon-La Choi, Cheol-Keun Park, Young Kee Shin

**Affiliations:** ^1^ College of Pharmacy, Kyungpook National University, Daegu, Korea; ^2^ Research Institute of Pharmaceutical Sciences, College of Pharmacy, Kyungpook National University, Daegu, Korea; ^3^ Laboratory of Molecular Pathology and Cancer Genomics, College of Pharmacy, Seoul National University, Seoul, Korea; ^4^ Tumor Microenvironment Global Core Research Center, Seoul National University, Seoul, Korea; ^5^ R&D center, ABION Inc., Guro-gu, Seoul, Korea; ^6^ Gencurix, Inc., Guro-gu, Seoul, Korea; ^7^ Laboratory of Cancer Genomics and Molecular Pathology, Samsung Medical Center, Sungkyunkwan University School of Medicine, Seoul, Korea; ^8^ The Center for Anti-cancer Companion Diagnostics, Institutes of Entrepreneurial BioConvergence, Seoul National University, Seoul, Korea; ^9^ Department of Pathology, Gachon University Gil Medical Center, Incheon, Korea; ^10^ Department of Pathology, Stanford University School of Medicine, Stanford, California, United States of America; ^11^ Department of Pathology and Translational Genomics, Samsung Medical Center, Sungkyunkwan University School of Medicine, Seoul, Korea; ^12^ Department of Health Sciences and Technology, SAIHST, Sungkyunkwan University, Seoul, Korea; ^13^ Department of Molecular Medicine and Biopharmaceutical Sciences, Graduate School of Convergence Science and Technology, Seoul National University, Seoul, Korea

**Keywords:** gastric cancer, DNA copy number alterations, potential cancer-promoting genes, ERBB2, MET

## Abstract

Gastric cancer (GC), one of the most common cancers worldwide, has a high mortality rate due to limited treatment options. Identifying novel and promising molecular targets is a major challenge that must be overcome if treatment of advanced GC is to be successful. Here, we used comparative genomic hybridization and gene expression microarrays to examine genome-wide DNA copy number alterations (CNAs) and global gene expression in 38 GC samples from old and young patients. We identified frequent CNAs, which included copy number gains on chromosomes 3q, 7p, 8q, 20p, and 20q and copy number losses on chromosomes 19p and 21p. The most frequently gained region was 7p21.1 (55%), whereas the most frequently deleted region was 21p11.1 (50%). Recurrent highly amplified regions 17q12 and 7q31.1-7q31.31 harbored two well-known oncogenes: *ERBB2* and *MET*. Correlation analysis of CNAs and gene expression levels identified *CAPZA2* (co-amplified with *MET*) and genes *GRB7*, *MIEN1*, *PGAP3*, and *STARD3* (co-amplified with *ERBB2*) as potential candidate cancer-promoting genes (CPGs). Public dataset analysis confirmed co-amplification of these genes with *MET* or *ERBB2* in GC tissue samples, and revealed that high expression (except for *PGAP3*) was significantly associated with shorter overall survival. Knockdown of these genes using small interfering RNA led to significant suppression of GC cell proliferation and migration. Reduced GC cell proliferation mediated by *CAPZA2* knockdown was attributable to attenuated cell cycle progression and increased apoptosis. This study identified novel candidate CPGs co-amplified with *MET* or *ERBB2*, and suggests that they play a functional role in GC.

## INTRODUCTION

Gastric cancer (GC) is one of the most common malignancies and the third most prevalent cause of cancer death worldwide [1]. Although curative therapy with surgical resection is possible at the early stages, most patients are diagnosed with advanced disease, which has a poor prognosis [2]. The 5 year survival rate of patients with advanced GC is only 25% to 35% [3, 4], and chemotherapy remains the main treatment. Therefore, appropriate therapies for advanced or metastatic GC are urgently needed. However, only two therapeutic antibodies, trastuzumab (which targets HER2) and ramucirumab (which targets VEGFR2), have been approved for use as treatments for GC. Clinical trials show that, compared with chemotherapy alone, addition of trastuzumab to chemotherapeutic regimens improves patient survival [5–7], and that ramucirumab monotherapy, or ramucirumab in combination with chemotherapy, confers survival benefits on advanced GC patients that have received prior treatments [8, 9]. Despite the proven efficacy of trastuzumab against HER2-overexpressing GC, trastuzumab resistance remains problematic. Accordingly, a better understanding of the molecular alterations that occur during GC progression is required if we are to identify novel and promising therapeutic targets for GC.

Early-onset GC, defined as GC occurring at age 45 years or younger, is clinicopathologically, histologically, and molecularly distinct from conventional GC [10]. According to Laurén's classification, there are two main histological subtypes: diffuse and intestinal. Diffuse-type GC is more common in young patients and is multifocal, hereditary, and infrequently accompanied by intestinal metaplasia, whereas intestinal-type GC occurs more frequently in older patients and follows multifocal atrophic gastritis [10]. Given that genetic factors are more important in young patients, who are generally less exposed to environmental carcinogens than older patients, genomic comparison of early-onset GC with GC in older patients may help to unravel the genetic changes that occur during gastric carcinogenesis [10].

Analyses of genomic DNA copy number alterations (CNAs) and global gene expression have been used extensively to identify candidate driver genes or cancer-promoting genes (CPGs) as therapeutic targets or novel biomarkers for GC [11–15]. The Cancer Genome Atlas (TCGA) project recently described four major molecular subtypes of gastric adenocarcinoma based on integrated analysis of genomic/epigenomic alterations identified in 295 primary gastric adenocarcinomas [16]. These subtypes highlight the heterogeneity of GC. Another recent study identified molecular subtypes of GC associated with clinical outcomes; these subtypes differ from those in the TCGA classification [17]. However, most of these studies are limited to identification of candidate genes using genomic approaches without subsequent validation; further functional validation of candidate genes is required if they are to become potential therapeutic targets.

Here, we identified frequent genome-wide CNAs in 38 GC tissue samples and demonstrated differences in CNAs between GC samples from young and old patients. Furthermore, we identified novel, potential candidate CPGs based on integrated analysis of genome-wide CNAs and gene expression profiling, and validated their amplification and expression in TCGC data. The association between gene expression and patient outcome was also assessed using a public dataset, and we examined the function of these genes in GC cell lines using small interfering RNA (siRNA).

## RESULTS

### Array-based DNA CNA analysis of GC tissues

First, to identify DNA CNAs in the GC genome we performed high-density oligonucleotide array comparative genomic hybridization (aCGH) of genomic DNA from 40 GC samples obtained from 19 old (O1 to O19) and 21 young patients (Y1 to Y21). However, two samples (O12 and Y14) were excluded due to poor microarray data quality; therefore, 38 patients (18 old and 20 young) were included in the analysis. Clinical details and data about sample quality are provided in Supplementary Table 1. The median age of the old patients was 76.5 years (range, 70–86), and that of the young patients was 34 years (range, 30–38). The cancers in the old and young patient groups were of different histological types (*P* = 0.003); with one exception, all of the old patients had tubular adenocarcinoma and intestinal-type cancers, whereas 11 of the 20 young patients had poorly cohesive carcinoma and diffuse-type cancer.

**Figure 1:**
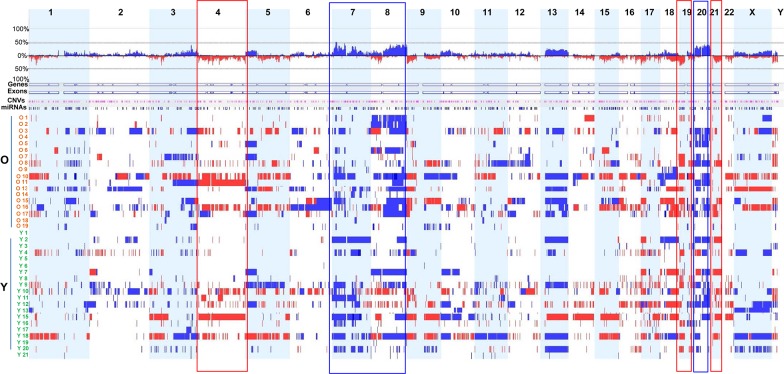
Genome-wide DNA CNAs across 38 GC samples Frequency landscapes of genome-wide DNA CNAs across 38 GC tissues from old and young patients. The vertical axis (between upper and lower lines for 100% frequency) represents DNA CNA frequency at the corresponding chromosomal positions. Blue and red indicate gain and loss, respectively, of genomic DNA copy number. Rectangular boxes with blue and red boundary lines indicate genomic regions showing high frequency copy number gains and losses, respectively. Samples from old patients and young patients are designated O1 to O19 and Y1 to Y21, respectively.

We found frequent and recurrent CNA regions among the 38 samples; these included gains on chromosomes 3q, 7p, 8q, 20p, and 20q (frequency ≥ 40%) and losses on chromosomes 19p and 21p (frequency ≥ 40%) (Figure 1 and Supplementary Table 2). The most frequently gained region was 7p21.1 (frequency, 55%), which harbors *AGMO*, *ISPD*, *LOC100506025*, *MEOX2*, *SOSTDC1*, and *HDAC9*. The second most frequently gained region was 7p12.2 (frequency, 50%), which harbors *GRB10*. In addition, the following regions showed frequent gains: 8q21.11–8q21.12, 8q22.3, 8q24.21, 8q24.22, 8q24.3, 20q12, and 20q13.2 (frequency, 45%); 3q26.2, 8q23.1–q23.3, 8q23.3–24.11, 8q24.21–q24.22, 20q13.2, 20q13.32, and 20q13.33 (frequency, 42.5%); and 7p15.2–7p15.1, 8p21.3, 20p12.3–p12.2, 20q11.21, 20q11.23, 20q13.12, and 20q13.13 (frequency, 40%).

Frequent copy number losses were observed at 21p11.1 (frequency, 50%), 19p13.2, and 19p13.3 (frequency, 40%). Other regions showing frequent copy number losses included 1p35.3, 3p21.31, 4q12, and 17p13.3 (frequency, 37.5%).

Of the CNAs found among the 38 samples, highly amplified (or high copy number gain) and homozygous deletion regions were also analyzed. Regions with high copy number gain were observed on chromosomes 3, 7, 8, 10, 11, 12, 17, 19, and 20, with the highest gain detected on chromosome 17 (median log_2_ ratio > 4.5) (Supplementary Table 3). In particular, highly amplified regions contained well-known oncogenes such as *EGFR* (7p11.2; O17), *ERBB2* (17q12; O13, O16, Y4, Y7, Y18), *FGFR2* (10q26.12-q26.13; Y21), *MET* (7q31.1-q31.31; Y4, Y15), *MYC* (8q24.21; O19), and *PIK3CA* (3q26.32-q26.33, O17) (Supplementary Table 3). Other oncogenes or cancer-associated genes such as *CCNE1* (19q12; O15), *CD44* (11p13; Y21), *CDK6* (7q21.2; Y11), *GATA4* (8p23.1-p22; O19), and *GATA6* (18q11.2; O17) were also located in regions with high copy number gains. Importantly, the regions including the oncogenes *ERBB2* and *MET* were recurrent and highly amplified (Supplementary Table 3). The region in which *ERBB2* is located also contains genes such as *GRB7*, *MIEN1* (also known as *C17orf37*), *MIR4728*, *PGAP3* (also known as *PERLD1*), *PNMT*, *PPP1R1B*, *STARD3*, and *TCAP*. The chromosome region containing *MET*, 7q31.1-7q31.31, also harbors *ASZ1*, *C7orf60*, *CAPZA2*, *CAV1*, *CAV2*, *CFTR*, *CTTNBP2*, *FOXP2*, *GPR85*, *LOC401397*, *MDFIC*, *MIR3666*, *PPP1R3A*, *ST7*, *ST7-AS1*, *ST7-AS2*, *ST7-OT3*, *ST7-OT4*, *TES*, *TFEC*, *TMEM168*, and *WNT2*.

Genomic regions harboring homozygous deletions included 5q35.2-5q35.3, 7q35, 9p21.3, 17p12, 17p13.3, 21p11.1, Yp11.2, and Yq11.21 (Supplementary Table 3). Notably, the genomic region 9p21.3 (O10) includes two well-known tumor suppressor genes: *CDKN2A* (also known as *INK4A* or *P16INK4A*) and *CDKN2B* (also known as *INK4B* or *P15INK4B*) (Supplementary Table 3). Intriguingly, this *CDKN2A*-*CDKN2B* cluster contains *CDKN2B-AS1* (also known as *ANRIL*), a non-coding RNA transcribed in the antisense orientation of *CDKN2A* and *CDKN2B*. This non-coding RNA interacts with polycomb repressive complexes 1 and 2, leading to epigenetic repression of other tumor suppressor genes within the cluster, thereby facilitating tumor growth [18]. The 9p21.3 region also harbors the cancer-associated gene *MTAP*, which is adjacent to the *CDKN2A-CDKN2B* cluster. A previous study [19] reported that genomic deletion contributes to reduced *MTAP* expression in GC, and that downregulation of *MTAP* is associated with a poor prognosis. This study also demonstrated a tumor suppressive role for *MTAP* in cell growth and invasion, suggesting that it plays a role in GC progression. Other genomic regions with homozygous deletions also contained genes (*MAP2K4* and *miR-744* in 17p12 [Y15] and *RPH3AL* in 17p13.3 [Y12], respectively) that function as tumor suppressors or potential tumor suppressors in other cancers (Supplementary Table 3) [20–22].

We also compared DNA CNAs between two different histopathological subtypes of GC. CNAs in intestinal-type and tubular adenocarcinoma were significantly more frequent (*n* = 26) than those in diffuse-type and poorly cohesive carcinoma (*n* = 12) (*P* = 0.006). Notably, we found that, among the 38 GC samples, seven from patients with diffuse-type and/or signet ring cell carcinoma (O18, Y1, Y5, Y6, Y17, Y19, and Y21) harbored relatively few CNAs (Figure 1 and Supplementary Table 1). The high frequency of CNAs in intestinal-type and tubular adenocarcinoma identified in this study is consistent with a recent TCGA study showing that intestinal-type GC is more susceptible to frequent genomic alterations than other types of GC [16].

### Comparison of genome-wide DNA CNAs in young and old patients with GC

**Figure 2:**
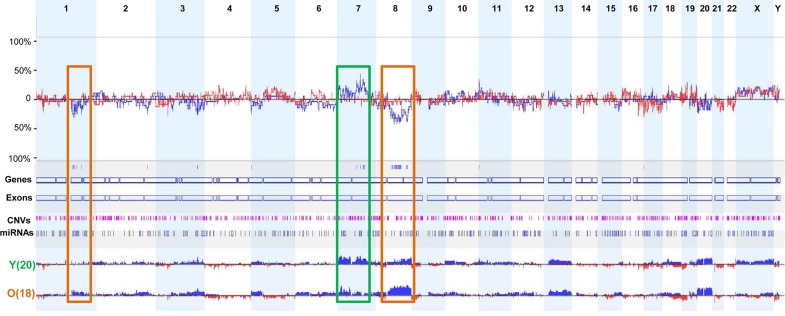
Comparison of the frequencies of DNA CNAs between young and old patients with GC Three major genomic regions showed distinct differences in the frequency of DNA CNAs in young and old patients with GC. Rectangular boxes with brown boundaries indicate regions in which copy number gains were more frequently observed in old patients, whereas the rectangular box with green boundaries indicates the region in which more frequent copy number gains were found in young patients.

Next, we analyzed differences in DNA CNAs between young and old patients with GC according to genome-wide CNA frequency. Six young patients with diffuse-type and/or signet ring cell carcinoma (Y1, Y5, Y6, Y17, Y19, and Y21) had relatively few CNAs (Figure 1 and Supplementary Table 1). We identified three major regions of difference (1p12-1q31.1, 7q21.11-7q31.32, and 8q12.1-8q24.22) between the two groups. Compared with young patients, old patients with GC demonstrated more frequent copy number gains in regions 8q12.1-8q24.22 and 1p12-1q31.1, whereas copy number gains in the region 7q21.11-7q31.32 was more frequent in young patients (Figure 2).

Interestingly, the region 8q24.21, which harbors oncogene *MYC*, showed more frequent gains in old patients (12/18, 66.7%) than in young patients (6/20, 30%) (Supplementary Table 4). The other genomic region, 1p12-1q31.1, contains the oncogene *CHD1L*, which is an independent, negative prognostic factor for GC [23]. Six of the old patients showed copy number gains in the region 1q21.1, which contains *CHD1L*, whereas only two young patients had copy number gains in this region (Supplementary Table 4). By contrast, oncogenes *CDK6* and *MET*, located in region 7q21.11-7q31.32, showed more frequent copy number gains in young patients than in old patients. Of note, high copy number gains of *CDK6* and *MET* were found only in young patients (Supplementary Table 4). These data indicate that *CDK6* and *MET* may be associated with early-onset GC.

### Analysis of the correlation between DNA CNAs and gene expression to identify candidate CPGs in GC

To identify potential candidate CPGs in GC, we performed correlation analysis of CNAs and gene expression using an integrated approach based on aCGH and gene expression microarray data. We then calculated Pearson's correlation coefficient between DNA CNAs and gene expression for each gene and identified 2,060 genes that showed a significant positive correlation (correlation coefficient > 0.5, false discovery rate < 5%) (Supplementary Table 5). This group included a number of well-known oncogenes, including *ERBB2*, *PIK3CA*, *MET*, *CCNE1*, *EGFR*, and *CDK6*, which supports the hypothesis that overexpressed genes associated with gene amplification are potential candidate driver genes or CPGs.

Among the 2,060 correlated genes, the top 20 showing the highest positive correlation between copy number gain and expression were *LSM12* (correlation coefficient, 0.9593), *SMARCE1* (0.9327), *TRIP12* (0.9164), *CLNS1A* (0.9137), *POP4* (0.9119), *ERBB2* (0.9044), *MSL-1* (0.8988), *PSMD3* (0.8936), *PPM1D* (0.8905), *C19orf12* (0.8867: 0), *COPS5* (0.8823), *C19orf2* (0.8797), *MIEN1* (0.8773), *SEC61G* (0.8713), *CASC3* (0.8713), *UQCRFS1* (0.8687), *ARIH1* (0.8677), *KRTAP3-3* (0.8661), *STARD3* (0.8626), and *CNOT7* (0.854).

Notably, the copy number gain of *ERBB2*, *MIEN1* (migration and invasion enhancer 1), *STARD3* (StAR related lipid transfer domain containing 3), *PGAP3* (post-GPI attachment to proteins 3), and *GRB7* (growth factor receptor bound protein 7), all of which reside in the *ERBB2* amplicon (17q12), and that of *CAPZA2* (capping actin protein of muscle Z-line alpha subunit 2) and *MET*, which are located in the *MET* amplicon (7q31.1-7q31.31), showed a strong correlation (correlation coefficient > 0.7) with high gene expression (Supplementary Table 5). These findings suggest that genes co-amplified with *ERBB2* or *MET* are potential CPGs for GC.

### Validation of DNA CNAs, gene expression, and clinical significance of genes co-amplified with *MET* or *ERBB2* in public dataset

**Figure 3:**
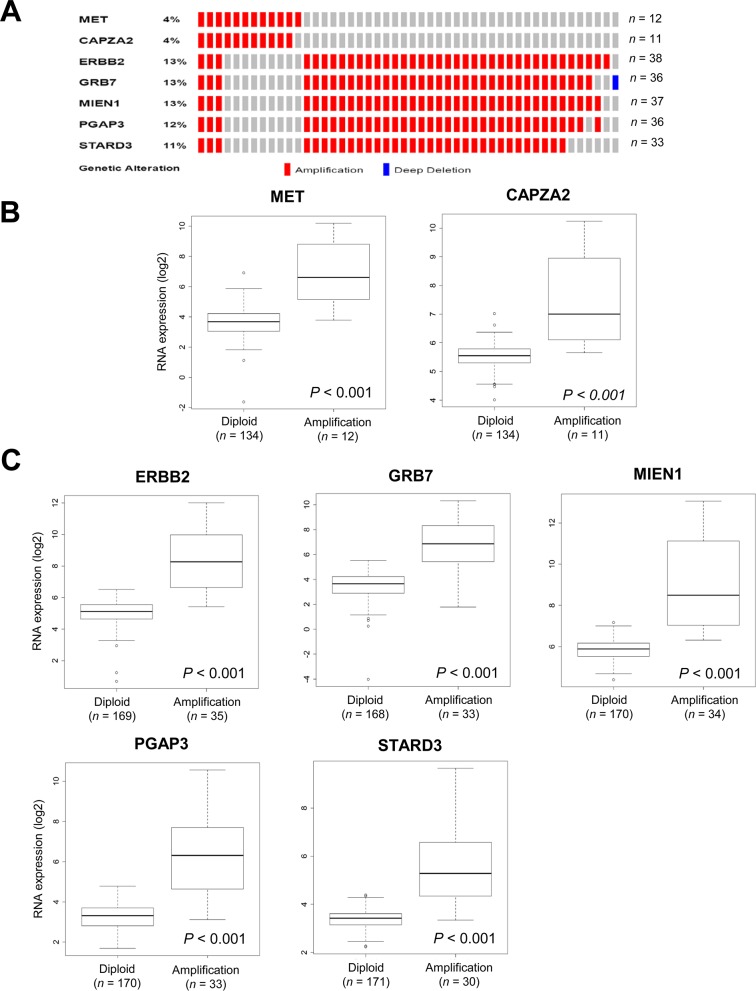
Validation of CNAs and gene expression of genes co-amplified with *MET* or *ERBB2* in TCGA data **(A)** OncoPrint of CNAs in *CAPZA2* and genes co-amplified with *ERBB2* in 295 GC samples based on TCGA data [16]. **(B, C)** Box plot showing the association between their mRNA levels and gene amplification. The horizontal line within the box indicates the median, boundaries of the box indicate the 25^th^ and 75^th^ percentile, and the whiskers indicate the highest and lowest values of the results. OncoPrint was generated using the cBioPortal (http://www.cbioportal.org/). Statistical differences between the two groups were assessed using the Mann-Whitney test. The number of patients for each group (in parentheses) is provided.

We further validated the DNA CNAs and gene expression of candidate CPGs using TCGA data from 295 GC tissues [16]. In agreement with our findings in the 38 GC samples, frequent amplification of *CAPZA2*, or four genes co-amplified with *ERBB2* (*MIEN1*, *GRB7*, *PAGP3*, and *STARD3*), was observed (Figure 3A); expression of these genes was significantly higher in GC samples in which they were amplified than in samples in which they were not (*P* < 0.001) (Figure 3B and 3C).

**Figure 4:**
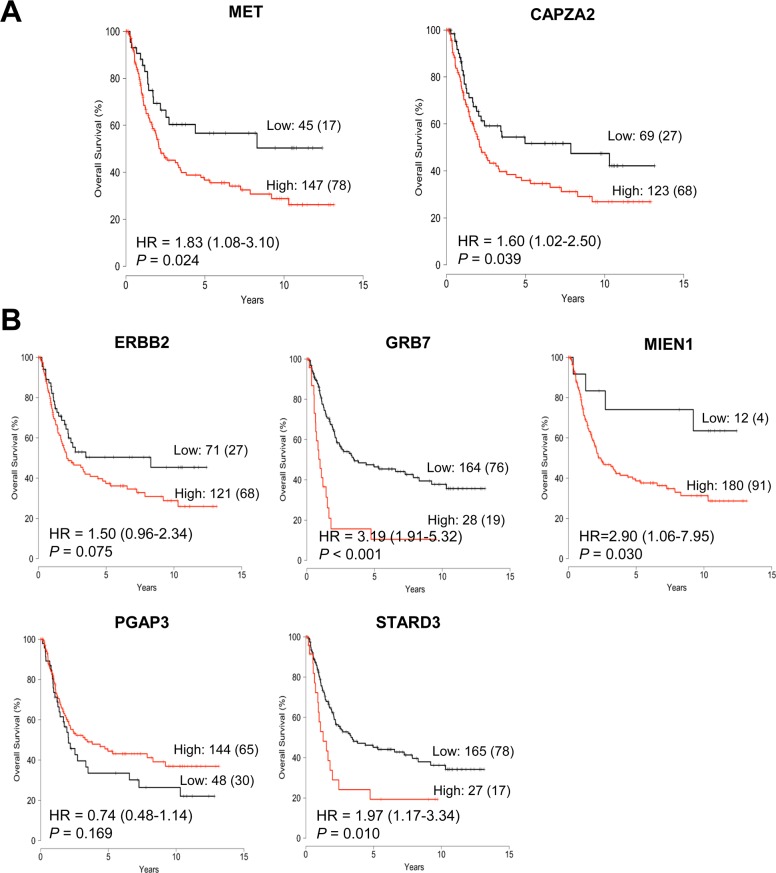
Association between expression of genes co-amplified with *MET* or *ERBB2* and overall survival in patients with GC Kaplan-Meier plot of overall survival (OS) for GC patients stratified by gene expression of **(A)** *CAPZA2* and *MET*, and **(B)** *ERBB2* and genes co-amplified with *ERBB2* (*GRB7*, *MIEN1*, *PGAP3*, and *STARD3*). Patients were divided into low expression and high expression groups according to cut-off value, and statistical differences in survival between the two groups were tested using the log-rank test. The number of patients in each group and the number of events (death) are provided. HR: hazard ratio.

Next, we analyzed the association between their expression and patient outcome using a public dataset from the Gene Expression Omnibus (GEO). In agreement with previous studies [24, 25], we found that higher expression of *MET* or *ERBB2* correlated with an unfavorable prognosis (Figure 4). Patients with high *MET* expression had significantly shorter overall survival (OS) than those with low *MET* expression (*P* = 0.024). High *ERBB2* expression showed a trend toward shorter OS (*P* = 0.075). Importantly, high *CAPZA2* expression was significantly associated with increased risk of death (hazard ratio [HR], 1.60; 95% confidence interval [CI], 1.02–2.50; *P* = 0.039). Among the genes co-amplified with *ERBB2*, high expression of three genes (*PGAP3* was an exception) showed a significant association with increased risk of death (HR = 3.19, *P* < 0.001 for *GRB7*; HR = 2.90, *P* = 0.030 for *MIEN1*; and HR = 1.97, *P* = 0.010 for *STARD3*). Taken together, these data confirm frequent amplification of candidate CPGs and their correlation with high gene expression in a large number of GC tissue samples. Moreover, the results showed that high expression of the candidate CPGs identified herein is significantly associated with poor prognosis of GC patients, suggesting that they have prognostic significance in GC.

### Effect of genes co-amplified with *MET* or *ERBB2* on the proliferation and migration of GC cells

Based on the clinical significance of candidate CPGs in the public dataset, we investigated whether they play a functional role in GC cells. First, we analyzed DNA CNA and expression of genes co-amplified with *MET* or *ERBB2* in 14 GC cell lines using Human Exonic Evidence Based Oligonucleotide (HEEBO) microarray data. Fourteen GC cell lines were classified into two histological types, intestinal-type (AGS, MKN1, MKN28, MKN74, NCI-N87, SNU216, and SNU719) and diffuse-type (KATOIII, SNU5, SNU484, SNU601, SNU620, SNU638, and SNU668), as described in our previous study [26]. HEEBO data revealed that *MET* and *CAPZA2* show high expression and high amplification in two diffuse-type GC cell lines: SNU620 and SNU5 (Supplementary Figure 1). NCI-N87 and SNU216 cells showed high expression and amplification of *ERBB2* (Supplementary Figure 2). Real-time polymerase chain reaction (PCR) data confirmed both gene amplification and high expression of these genes in the cell lines (Supplementary Figure 1 and 2).

**Figure 5:**
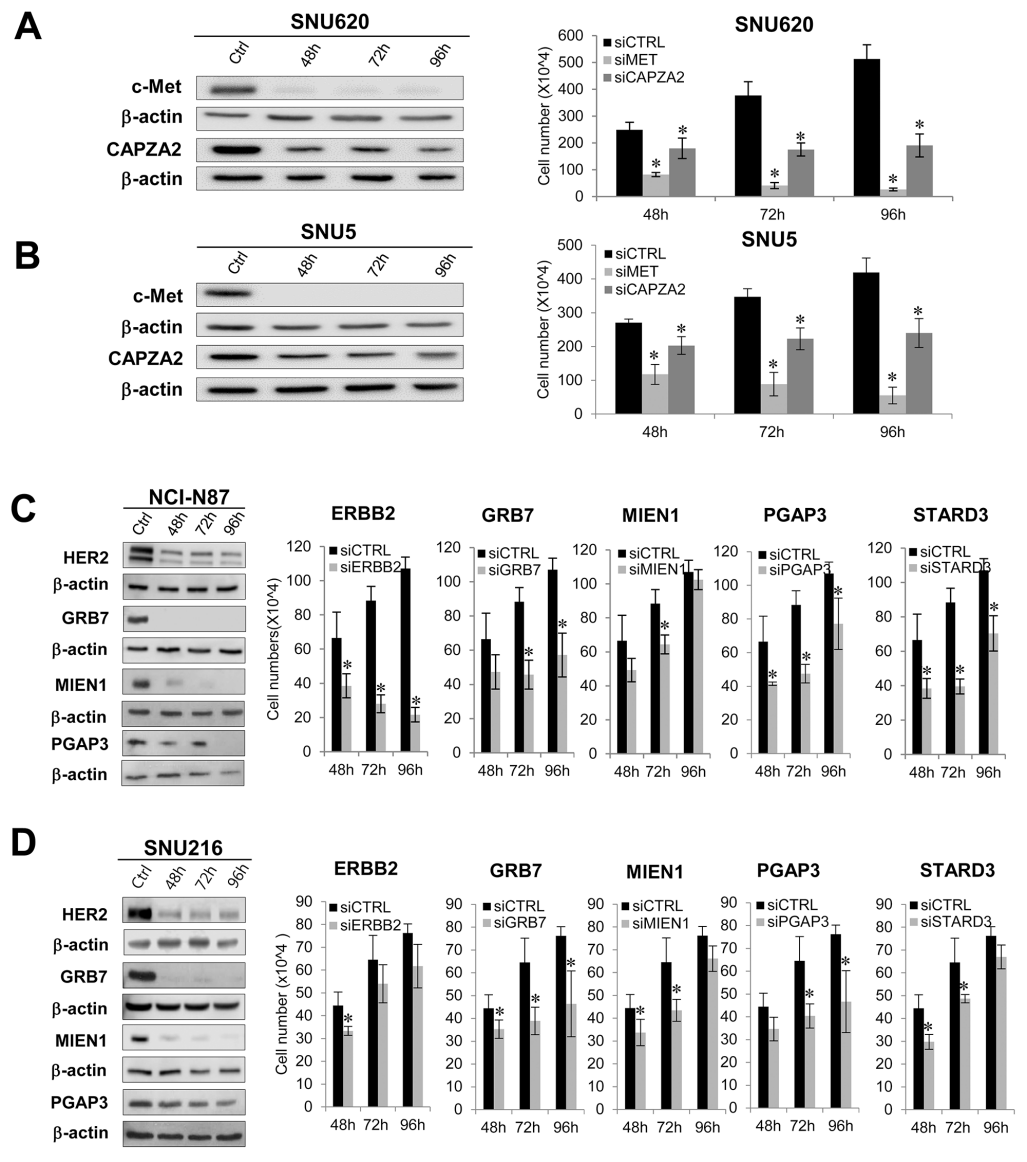
Effect of siRNA-mediated knockdown of genes co-amplified with *MET* or *ERBB2* on proliferation of GC cells **(A,B)** Effect of *MET* or *CAPZA2* knockdownon proliferation of diffuse-type GC cell lines SNU620 and SNU5. **(C,D)** Effect of knocking down *ERBB2*, *GRB7*, *MIEN1*, *PGAP3*, or *STARD3* on proliferation of intestinal-type NCI-N87 and SNU216 cells. The protein products of target genes before (ctrl) and after siRNA treatment were measured by western blotting in a time-dependent manner (48, 72, and 96 h). Depletion or marked reductions in target protein levels in siRNA-treated cells compared with before siRNA treatment were confirmed (the exception was STARD3). Significant knockdown of *STARD3* at the mRNA level was confirmed by qRT-PCR (data not shown). The number of viable control siRNA (siCTRL)- or target gene siRNA (siCAPZA2, siMET, siERBB2, siGRB7, siMIEN1, siPGAP3, siSTARD3)-transfected cells was measured at the indicated times after siRNA treatment. Data are presented as the mean ± standard deviation (SD) of three or four experiments. Mann-Whitney test, ^*^P < 0.05.

**Figure 6:**
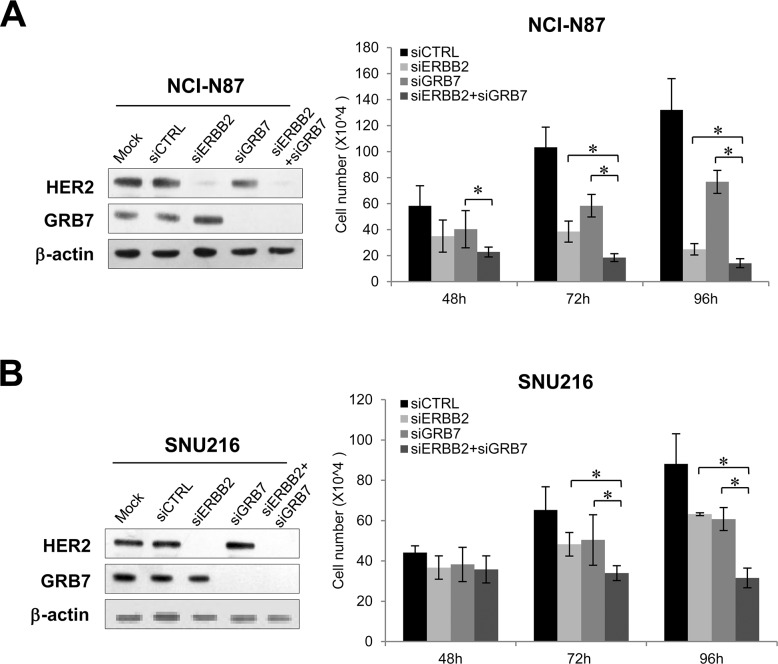
Effect of combined knockdown of *ERBB2* and *GRB7* on proliferation of GC cells Effect of combined knockdown of *ERBB2* and *GRB7* compared with single knockdown on proliferation of **(A)** NCI-N87 and **(B)** SNU216 cells. There was significant depletion of target protein levels in *ERBB2* siRNA- or *GRB7* siRNA-treated cells when compared with control siRNA-treated cells. The number of viable siRNA-transfected cells was measured at the indicated time points after siRNA treatment. Data are presented as the mean ± SD of three experiments. Mann-Whitney test, ^*^P < 0.05.

Next, we examined the effect of *MET* or *CAPZA2* knockdown on proliferation of GC cells. Compared with cells transfected with control siRNA, SNU620 and SNU5 cells transfected with *MET* or *CAPZA2* siRNA showed a reduced cell proliferation (Figure 5A and 5B), indicating that knockdown of these genes inhibits proliferation of GC cells. We also assessed the effect of knockdown of *ERBB2* or genes co-amplified with *ERBB2* on cell proliferation in NCI-N87 and SNU216 cells. The number of siRNA-transfected NCI-N87 cells was significantly lower than that of control siRNA-transfected cells (Figure 5C). Similar to the results in NCI-N87 cells, knockdown of these genes in SNU216 cells significantly inhibited their proliferation, even though the effect in this cell line was weaker than that in NCI-N87 cells (Figure 5D). Of note, among genes co-amplified with *ERBB2*, *GRB7* knockdown caused the most significant inhibition of cell proliferation in both GC cell lines; therefore, we investigated the effect of knocking down both *ERBB2* and *GRB7* on cell proliferation. The results demonstrated that, compared with knockdown of *ERBB2* or *GRB7* alone, combined knockdown further inhibited cell proliferation (Figure 6). These results suggest that the anti-cancer effect of HER2*-*targeted therapy in GC can be further potentiated by addition of a *GRB7*-targeting agent.

The effect of gene knockdown on migration of GC cells was also assessed; we found that, compared with control siRNA, *CAPZA2* siRNA significantly suppressed migration of SNU620 cells, although this inhibitory effect was lower than that observed for *MET* siRNA (Supplementary Figure 3). Knockdown of genes co-amplified with *ERBB2* also led to significant inhibition of GC cell migration (Supplementary Figure 3). Taken together, these data suggest that *CAPZA2* and genes co-amplified with *ERBB2* affect proliferation and migration of GC cells.

### Mechanism underlying reduced cell proliferation by knockdown of genes co-amplified with *MET* or *ERBB2* in GC cells

**Figure 7:**
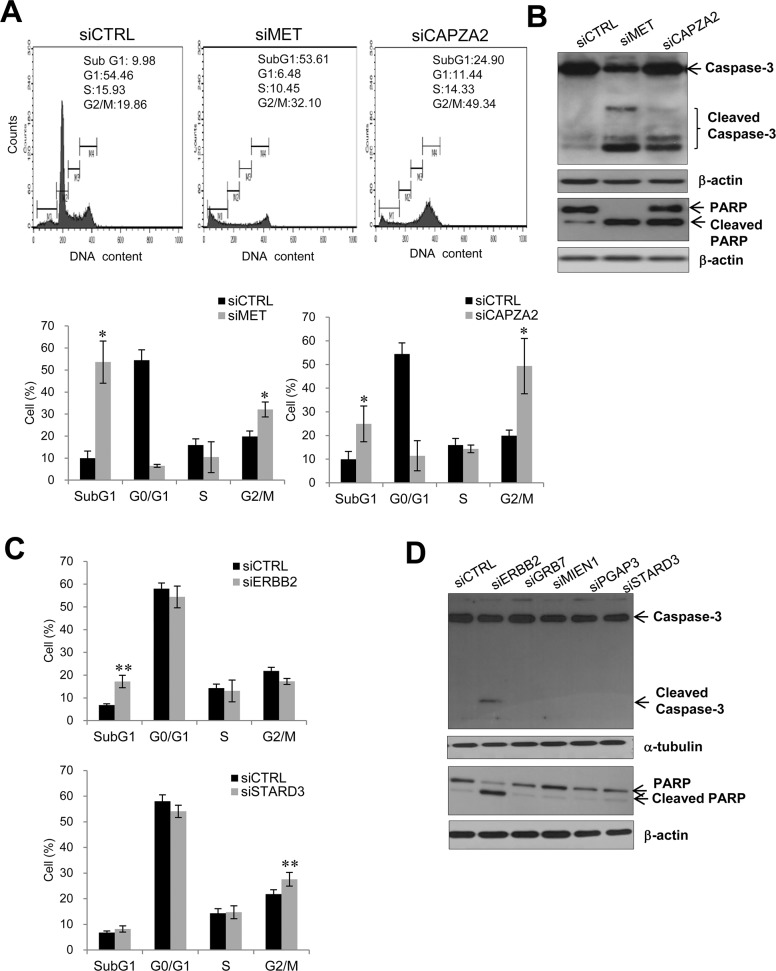
Effect of siRNA-mediated knockdown of genes co-amplified with *MET* or *ERBB2* on cell apoptosis or cell cycle progression **(A)** Cell cycle analysis using flow cytometry and **(B)** western blot analysis of apoptosis markers in SNU620 cells treated with *CAPZA2* or *MET* siRNA. **(C)** Cell cycle analysis using flow cytometry and **(D)** western blot analysis of apoptosis markers in NCI-N87 cells treated with siRNAs targeting *ERBB2* or genes co-amplified with *ERBB2* (*GRB7*, *MIEN1*, *PGAP3*, or *STARD3*). For cell cycle analysis, the percentage of cells at each phase is shown as the mean ± SD of at least three experiments. Mann-Whitney test, ^*^P < 0.05, ^**^P < 0.01.

To investigate the molecular mechanism underlying the growth inhibitory effect of knocking down genes co-amplified with *MET* or *ERBB2* in GC cells, we performed cell cycle analysis using flow cytometry. The percentage of *CAPZA2* siRNA-treated SNU 620 cells in subG1 (24.90% ± 7.58% *vs*. 9.98% ± 7.58%, *P* = 0.034) and G2/M (49.34% ± 11.67% *vs*. 19.86% ± 2.43%, *P* = 0.034) phase was significantly higher than that of control siRNA-treated cells (Figure 7A), suggesting that *CAPZA2* knockdown-mediated reduction in cell proliferation is attributable to both increased apoptosis and inhibition of cell cycle progression. Moreover, western blot analyses to detect cleaved PARP and caspase-3 proteins confirmed increased apoptosis in *CAPZA2* knockdown cells (Figure 7B). Higher G2/M accumulation (2.48-fold *vs*. 1.62-fold) and less apoptosis induction (2.49-fold *vs*. 5.37-fold) was observed in *CAPZA2* knockdown cells than in *MET* siRNA-treated cells, respectively. These results illustrate that attenuated cell cycle progression rather than profound apoptosis induction plays the major role in reduced GC cell proliferation induced by *CAPZA2* knockdown compared with *MET* knockdown.

Transfection of NCI-N87 cells with *ERBB2* siRNA led to a significant increase in the percentage of cells in subG1 phase when compared with control siRNA treatment (2.52-fold, *P* = 0.006) (Figure 7C). Cleaved caspase-3 and PARP were also found in *ERBB2* siRNA-treated cells (Figure 7D). These data indicate that reduced cell proliferation induced by *ERBB2* knockdown is due to increased apoptosis. By contrast, NCI-N87 cells transfected with siRNAs targeting genes co-amplified with *ERBB2* showed no increase in the subG1 fraction; nor was there an increase in cleaved PARP and caspase-3 on western blots. There was a slightly higher percentage of cells in G2/M in *STARD3* knockdown cells than in control siRNA-treated cells (1.26-fold, *P* = 0.006) (Figure 7C). However, knocking down *GRB7*, *MIEN1*, or *PGAP3* did not affect cell cycle progression (data not shown). These results suggest that reduced cell proliferation induced by *GRB7*, *MIEN1*, or *PGAP3* silencing is not related to attenuated cell cycle progression or cell death; therefore, other yet-to-be identified mechanisms are involved.

### DNA CNAs of genes co-amplified with *MET* or *ERBB2* in various cancer types

**Figure 8:**
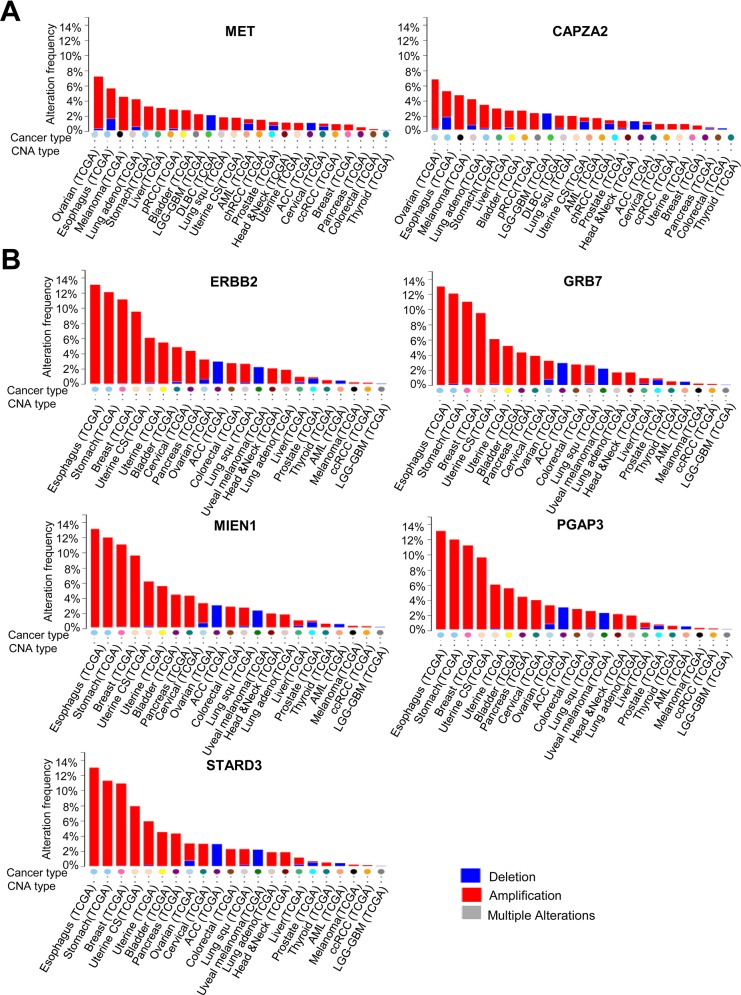
DNA CNA frequency of genes co-amplified with *MET* or *ERBB2* in diverse cancer types **(A)** DNA CNA frequency of *MET* and *CAPZA2*, and **(B)** DNA CNA frequency of *ERBB2* and genes co-amplified with *ERBB2* (calculated for diverse cancer types by analysing the TCGA data available at cBioPortal). ACC; Adrenocortical carcinoma, AML; Acute myeloid leukemia, chRCC; Kidney chromophobe, DLBC; Diffuse large B-cell lymphoma, LGG-GBM; Merged cohort of low-grade gliomas and glioblastoma, pRCC; Kidney renal papillary cell carcinoma, Uterine CS; Uterine carcinosarcoma.

The frequency of DNA CNA in genes co-amplified with *MET* or *ERBB2* in various cancer types (based on TCGA data) was examined using the cBioPortal (http://www.cbioportal.org/). The results showed that genes co-amplified with *MET* or *ERBB2* also show high amplification frequency in cancers other than GC. A high frequency of *CAPZA2* amplification was found in ovarian cancer, esophageal cancer, melanoma, and lung adenocarcinoma, as well as stomach cancer (Figure 8A). Genes co-amplified with *ERBB2* (*GRB7*, *MIEN1*, *PGAP3*, and *STARD3*) exhibited the highest amplification frequency in esophageal cancer, followed by stomach, breast, uterine, and bladder cancer (Figure 8B).

## DISCUSSION

Although a recent TCGA study described the molecular characterization of GC based on genomic/epigenomic analyses of 295 primary gastric adenocarcinomas using six genome-wide analysis platforms, understanding of the molecular mechanisms underlying GC progression remains incomplete and little progress has been made in identifying driver genes or CPGs as therapeutic targets.

Here, we conducted aCGH analysis of 38 GC samples and found frequent CNAs, including copy number gains on chromosomes 3q, 7p, 8q, 20p, and 20q and copy number losses on chromosomes 19p and 21p. These findings agree with the results of previous reports on GC [16, 27, 28], which demonstrates the reliability of our aCGH data. In particular, we showed that the most frequent copy number gains occurred in regions 7p21.1 and 7p12.2, whereas the most frequent copy number losses occurred in 21p11.1. By contrast, the highest copy number gain among highly amplified regions (including chromosomes 3, 7, 8, 17, and 19) was detected on chromosome 17. Importantly, we found that highly amplified regions contained well-known oncogenes (*EGFR*, *ERBB2*, *FGFR2*, *MET*, *MYC*, and *PIK3CA*) and well-known tumor suppressor genes (*CDKN2A* and *CDKN2B*), which are located in the regions with homozygous deletions; these findings were consistent with the results of previous studies [27, 28].

Additionally, we identified three major regions that differed between young and old GC patients. Oncogenes *MYC* (8q24.21) and *CHD1L* (1q21.1) were found in the regions with more frequent gains in old patients. CHD1L expression was positively correlated with distant metastasis and poor prognosis in patients with GC [23], but little is known about its role in GC progression. Therefore, additional functional studies on this gene may clarify its possible utility as a therapeutic target for GC. Notably, we found that oncogenes *CDK6* (7q21.2) and *MET* (7q31.1-q31.31) were located in the region with more frequent gains in young patients. Moreover, recurrent high copy number gains of *MET* were observed only in young patients. These findings indicate that *MET* amplification may be associated with early-onset GC. Although high *MET* amplification and expression correlate with poor clinical outcomes in patients with GC [24, 29], the prognostic significance of *MET* amplification or expression in early-onset GC remains unclear. Given that diffuse-type GC is frequently found in young patients, our interpretation is supported by a recent study demonstrating the association between *MET* amplification and diffuse-type GC [29]. The study showed that, among 113 GC patients evaluable for *MET* amplification, the majority of *MET*-amplified samples were found in patients with diffuse-type GC, whereas only one intestinal sample was *MET*-amplified. However, our interpretation is limited by the small sample size, and further studies using large samples are required to support our findings. A study to elucidate the role of *MET* in early-onset GC progression will also be needed.

Analysis of the correlation between DNA CNAs and gene expression across the 38 GC samples identified *CAPZA2* co-amplified with *MET*, and genes co-amplified with *ERBB2* (*GRB7*, *MIEN1*, *PGAP3*, and *STARD3*), as potential candidate CPGs in GC. They were among the top-ranked genes showing a high positive correlation between gene amplification and expression. Although the well-known oncogenes *ERBB2* and *MET* are validated as therapeutic targets for GC [27, 30], little is known about the genes co-amplified with them.

We validated the genetic alterations in candidate CPGs identified in the present study and evaluated the clinical significance of their expression in GC tissue samples using public datasets. TCGA data analysis confirmed that they are frequently co-amplified with *MET* or *ERBB2* in GC tissues, and that their expression is significantly higher in amplified samples than in non-amplified samples. Importantly, high expression of these genes (except *PGAP3*) was significantly associated with shorter OS of GC patients, suggesting their prognostic significance in GC.

We also demonstrated the function of *CAPZA2* and genes co-amplified with *ERBB2* in GC cells. Knocking down these genes using siRNA suppressed GC cell proliferation and migration. Both cell apoptosis and G2/M cell arrest were responsible for suppressed cell proliferation after *CAPZA2* knockdown (similar results were obtained after *MET* knockdown). These results suggest that *CAPZA2* plays a role in GC progression by regulating apoptosis or cell cycle progression. By contrast, reduced GC cell proliferation after knockdown of genes co-amplified with *ERBB2* was not attributable to increased apoptosis. Moreover, apart from *STARD3*, attenuated cell cycle progression was not related to reduced cell proliferation after gene knockdown. These results are consistent with those of a previous study [31] showing that *STARD3* knockdown in breast cancer cells inhibited cell cycle progression and cell proliferation. Taken together, our data imply that other mechanisms are involved in the regulation of cell proliferation by *GRB7*, *MIEN1*, or *PGAP3*. To the best of our knowledge, the present study is the first to report the functional role of these genes in GC cells, although a few studies have reported the cancer-promoting role of *MIEN1* in breast [32], oral [33], and prostate cancer [34], and the involvement of *GRB7* or *STARD3* in breast cancer cell invasion, survival [35], and cell proliferation [31].

Furthermore, we showed for the first time that simultaneous knockdown of *ERBB2* or *GRB7* resulted in more potent inhibition of GC cell proliferation than knockdown of either gene alone. These results strongly suggest that *ERBB2* and *GRB7* may affect GC cells in a cooperative fashion, and that frequent co-amplification of these two genes in various cancer types might be essential for promoting cancer progression. This finding is also supported by the results of a recent study [36] illustrating the simultaneous and dramatic downregulation of *ERBB2*, *GRB7*, *PERLD1(PGAP3)*, and *STARD3* (all of which reside in the *ERBB2* amplicon) by β-catenin depletion in breast cancer cells, which provides evidence that these genes are likely co-regulated. Given that overcoming resistance to the anti-HER2 antibody is one of the major challenges to the successful treatment of GC, it is notable that combined treatment with a GRB7-targeting agent and an anti-HER2 agent is likely to potentiate the effect of HER2-targeted therapy; this may be a superior treatment strategy for advanced GC.

TCGA data analysis of various cancer types showed that genes co-amplified with *MET* or *ERBB2* had high amplification frequencies in other cancers. These TCGA data analyses may support the hypothesis that the co-amplified genes identified as potential CPGs for GC in this study play pro-oncogenic roles in other cancers as well, similar to *ERBB2* [37] and *MET* [38].

In conclusion, we found frequent genomic CNAs in GC tissues and distinct differences in genomic CNAs between old and young patients with GC. We also identified novel candidate CPGs for GC, including genes co-amplified with *MET* or *ERBB2*, using integrated analysis of genome-wide CNAs and gene expression data. Public data analysis validated co-amplification of these genes with *MET* or *ERBB2*, and revealed that their high expression is significantly associated with poor prognosis in GC. Furthermore, we demonstrated for the first time that knockdown of genes co-amplified with *MET* or *ERBB2* suppresses GC cell proliferation and migration, and that decreased cell proliferation by *CAPZA2* is related to decreased cell cycle progression and increased apoptosis, which implies a possible cancer-promoting role in GC. In addition, we showed that combined knockdown of *GRB7* and *ERBB2* inhibited cell proliferation to a greater extent than knockdown of either gene alone, suggesting that combined treatment, for example, simultaneously targeting co-amplified genes such as *GRB7* with *ERBB2*, may be more effective than HER2-targeted therapy alone for the treatment of GC.

## MATERIALS AND METHODS

### Patient samples

Frozen specimens were obtained from 60 patients with GC who had undergone gastric surgery or gastroscopy at the Samsung Medical Center (Seoul, Korea) between 2001 and 2007. The quality of 60 frozen GC tissues was assessed by histological and pathological evaluation. Of the 60 tissue samples, 45 tumor samples with more than 50% tumor lesions were used for nucleic acid extraction and microarray analyses. The study was approved by the institutional review board (IRB) of Samsung Medical Center and performed in accordance with the Declaration of Helsinki. The study was retrospective in nature; therefore, the requirement for informed consent was waived by the IRB. Patient information was anonymized and de-identified prior to analysis.

### Array-based CGH

Genomic DNA was extracted from 45 tissue samples using the DNeasy blood & tissue kit (Qiagen, Hilden, Germany) according to the manufacturer's instructions. Forty DNA samples that met the criteria were used for aCGH. aCGH analyses were performed on genomic DNA using Human Genome CGH Microarray Kit 44K (Agilent Technologies, Santa Clara, CA, USA). Labeling and hybridization were performed according to the manufacturer's protocol. In brief, 1.5 μg of sample DNA and 1.5 μg of sex-matched reference DNA (Human Genomic DNA, Promega, Madison, WI, USA) were digested with *Alu*I and *Rsa*I (Promega) for 24 h at 37°C. The digested DNA was labeled using an Agilent Genomic DNA Labeling Kit Plus (Agilent Technologies). Sample DNA was labeled with Cy5-deoxyuridine triphosphate, and reference DNA was labeled with Cy3-deoxyuridine triphosphate. After purification, equal amounts of sample and reference DNA were pooled and mixed with 50 μg of human Cot-1 DNA, dissolved in hybridization buffer, denatured, and hybridized to the aCGH at 65°C for 24 h. Glass slides were washed and scanned according to the manufacturer's instructions. All critical steps of the microarray experiment were performed in an ozone-free laboratory environment to safeguard dye stability.

Microarray images were analyzed using FEATURE EXTRACTION v10.1.1.1 (Agilent Technologies) with linear normalization (protocol CGH-v4_10_Apr08), and the resulting data were imported into the Nexus platform (v6.0) (BioDiscovery, Hawthorne, CA, USA). After the raw data were normalized, the log_2_ ratios of Cy5 (sample) to Cy3 (reference) were calculated. Chromosomal aberrations were classified as “gain” when the normalized log_2_ ratio was > 0.7 and as “loss” when the ratio was < -0.7. High level amplification (high copy number gain) was defined as a log_2_ ratio > 1.2, whereas homozygous deletion was defined as a log_2_ ratio < -1.2, based on the GISTIC (Genomic Identification of Significant Targets in Cancer) algorithm [39].

### Gene expression microarray

RNA samples were extracted using an RNeasy mini kit (Qiagen), and samples with low RNA quality (RNA integrity number < 6) were excluded from the gene expression microarray. Gene expression microarray experiments were performed using a Whole Human Genome Oligo Microarray kit (Agilent Technologies), and labeling and hybridization were carried out according to the manufacturer's protocol. In brief, 2 μg of sample RNA and 2 μg of universal human reference RNA (Agilent Technologies) were labeled using an Agilent Quick Amp Labeling Kit. Sample RNA was labeled with Cy5-deoxycytidine triphosphate, and reference RNA with Cy3-deoxycytidine triphosphate. Labeled cRNA was then purified using RNeasy® mini spin columns (Qiagen). Hybridization was performed using the Agilent Gene Expression Hybridization Kit; samples were hybridized by rotating at 10 rpm (65°C) for 17 h and then washed before scanning. Gene expression microarray slides were scanned with the microarray scanner and analyzed with FEATURE EXTRACTION v10.1.1.1. The resulting data were imported into Nexus (v6.0) for expression analysis.

### Public dataset analysis

The TCGA gastric adenocarcinoma dataset, including gene expression (RNA-seq V2) and CNA data [16], was obtained from cBioPortal (http://www.cbioportal.org/) [40, 41]. Gene expression values were transformed to log_2_ values, and CNA was determined using GISTIC 2.0 [42]. Cases displaying either normal copy number or high level amplification of the target gene were selected, and mRNA expression levels (according to amplification status) were presented as box plots.

For survival analysis, the GSE15459 dataset based on Affymetrix Human Genome U133 Plus 2.0 Array for 200 primary GC [43] was obtained from the GEO (https://www.ncbi.nlm.nih.gov/geo/). Of the 200 samples, data from 192 primary tumor samples, along with clinical information, were used for survival analysis. The patients were divided into low and high expression groups using a cut-off value that maximized the sum of sensitivity and specificity for predicting survival outcome. A patient was assigned to the “high expression” group when the expression value was higher than the cut-off value. Otherwise, the sample was categorized as “low expression”.

### GC cell lines

Fourteen GC cell lines (AGS, KATO III, MKN1, MKN28, MKN74, NCI-N87, SNU5, SNU216, SNU484, SNU601, SNU620, SNU638, SNU668, and SNU719) were obtained from the Korean Cell Line Bank (KCLB) (Seoul, Korea) and authenticated by short tandem repeat analysis. All cell lines were also tested for mycoplasma contamination. According to the protocol for cell culture provided by KCLB, AGS cells were grown in Kaighn's F12 medium, KATO III cells in IMDM medium, and all other cell lines in RPMI 1640 medium supplemented with 10% fetal bovine serum and 100 U/ml penicillin-streptomycin.

### HEEBO microarray

HEEBO microarray experiments using all 14 GC cell lines (AGS, KATO III, SNU5, SNU484, SNU620, SNU668, MKN28, SNU719, MKN1, MKN74, SNU601, SNU216, SNU638, and NCI-N87) were performed according to the manufacturer's protocol (https://www.microarray.org/sfgf/heebo.do).

### Quantitative real-time PCR

Genomic DNA was isolated from gastric cell lines using the DNeasy blood & tissue kit (Qiagen). Quantitative real-time PCR (qPCR) was performed using SYBR Green. Relative quantification of gene copy number was performed using the standard curve method. ΔΔCq values were calculated by comparing the Cq value of samples with the copy number of a control (albumin; ALB) located at 4q11-q13, followed by normalization to the calibrator (normal blood genomic DNA). Total RNA was extracted from GC cells using Trizol (Invitrogen, Carlsbad, CA, USA) and reverse-transcribed into cDNA using a SuperScript™ II First-Strand Synthesis System (Invitrogen). Quantitative reverse transcription-PCR (qRT-PCR) was performed in a LightCycler (Roche Applied Science, Mannheim, Germany) using primers and probes from the Universal Probe Library (Roche Applied Science). *PAPOLA* was used as a reference gene to normalize and quantify gene expression. The sequences of the qPCR and qRT-PCR primers used in the study are listed in Supplementary Table 6.

### Transfection with siRNA

siRNAs targeting the human genes *CAPZA2*, *ERBB2*, *GRB7*, *MET*, *MIEN1*, *PGAP3*, or *STARD3* were purchased from Dharmacon (Lafayette, CO, USA). Cells were transfected with siRNAs using the DharmaFECT™ transfection reagent (Dharmacon) according to the manufacturer's protocol. The final siRNA concentrations used for transfection were 12.5 nM or 25 nM (according to cell type). Cells were harvested for protein and mRNA analyses at 48, 72, and 96 h post-transfection.

### Western blotting

Cells were lysed using RIPA buffer containing a protease inhibitor (Roche, Mannheim, Germany), and western blotting was performed with 20–30 μg of protein, according to standard procedures. The following primary antibodies were used for immunoblotting: c-Met (Invitrogen; 37-0100), CAPZA2 (Proteintech, Chicago, IL, USA; 15948-1-AP), HER2/ErbB2 (Thermo Scientific, Lab Vision; MS-730), MIEN1 (Abnova, Taipei City, Taiwan; Hooo84299-B01), GRB7 (Santa Cruz Biotechnology, Santa Cruz, CA, USA; sc-607), and PGAP3 (Abcam, Cambridge, UK; ab81368). Antibodies specific for caspase-3 (Cell Signaling Technologies, Denver, MA, USA; #9662) and PARP (46D11, Cell Signaling Technology; #9532) were also used to detect apoptosis.

### Cell proliferation assay

To determine the effect of siRNA transfection on cell proliferation, cells were collected at 48, 72, and 96 h post-transfection and then vial cells were counted using a hemocytometer. Cell viability was assessed by trypan blue staining.

### Transwell migration assay

Migration was assessed using Costar transwell chambers with 8-mm diameter pores (Corning, NY, USA) as described previously [44]. Cells were synchronized with a double thymidine block. After incubation at 37°C for 10 h, migrated cells on the lower surface of the membrane were fixed with 4% paraformaldehyde and then stained with hematoxylin. Cells were counted in five randomly selected microscopic fields.

### Flow cytometry for cell cycle analysis

Cells transfected with siRNAs for 72 h or 96 h were harvested, washed with cold PBS, and fixed in 70% ethanol. Fixed cells were stained for 30 min with propidium iodide solution (BD Biosciences, San Diego, CA, USA) and analyzed by flow cytometry (FACSCalibur; BD Biosciences).

### Statistical analysis

Data from two groups were compared using the Mann-Whitney test. For survival analysis, survival curves were estimated using the Kaplan-Meier method, and statistical differences in the survival between the two groups were tested using the log-rank test. OS was defined as the time from the date of surgery to the date of death or last follow-up. All statistical analyses were performed using R3.2.0 (http://r-project.org) or SPSS23 statistic software for Windows (SPSS, Chicago, IL, USA). *P* < 0.05 was considered statistically significant. All *P* values were two-sided.

## SUPPLEMENTARY MATERIALS FIGURES AND TABLES















## Abbreviations

aCGH: array comparative genomic hybridization
CI: confidence interval
CNA: copy number alteration
CPG: cancer-promoting gene
GC: gastric cancer
GEO: Gene Expression Omnibus
GISTIC: Genomic Identification of Significant Targets in Cancer
HEEBO: Human Exonic Evidence Based Oligonucleotide
HR: hazard ratio
IRB: institutional review board
KCLB: Korean Cell Line Bank
OS: overall survival
qPCR: quantitative real-time PCR
qRT-PCR: quantitative real-time reverse transcription-PCR
SD: Standard deviation
siRNA: small interfering RNA
TCGA: The Cancer Genome Atlas.
