# Supplementary material for: Genes co-amplified with ERBB2 or MET as novel potential cancer-promoting genes in gastric cancer
Source: Oncotarget. 2017 Sep 21;8(54):92209–26. doi: 10.18632/oncotarget.21150 (PMC5696175; doi:10.18632/oncotarget.21150)
Supplement: Supplementary file 1 [file oncotarget-08-92209-s001.pdf]

Genes co-amplified with *ERBB2* or *MET* as novel potential cancer-promoting genes in gastric cancer

SUPPLEMENTARY MATERIALS

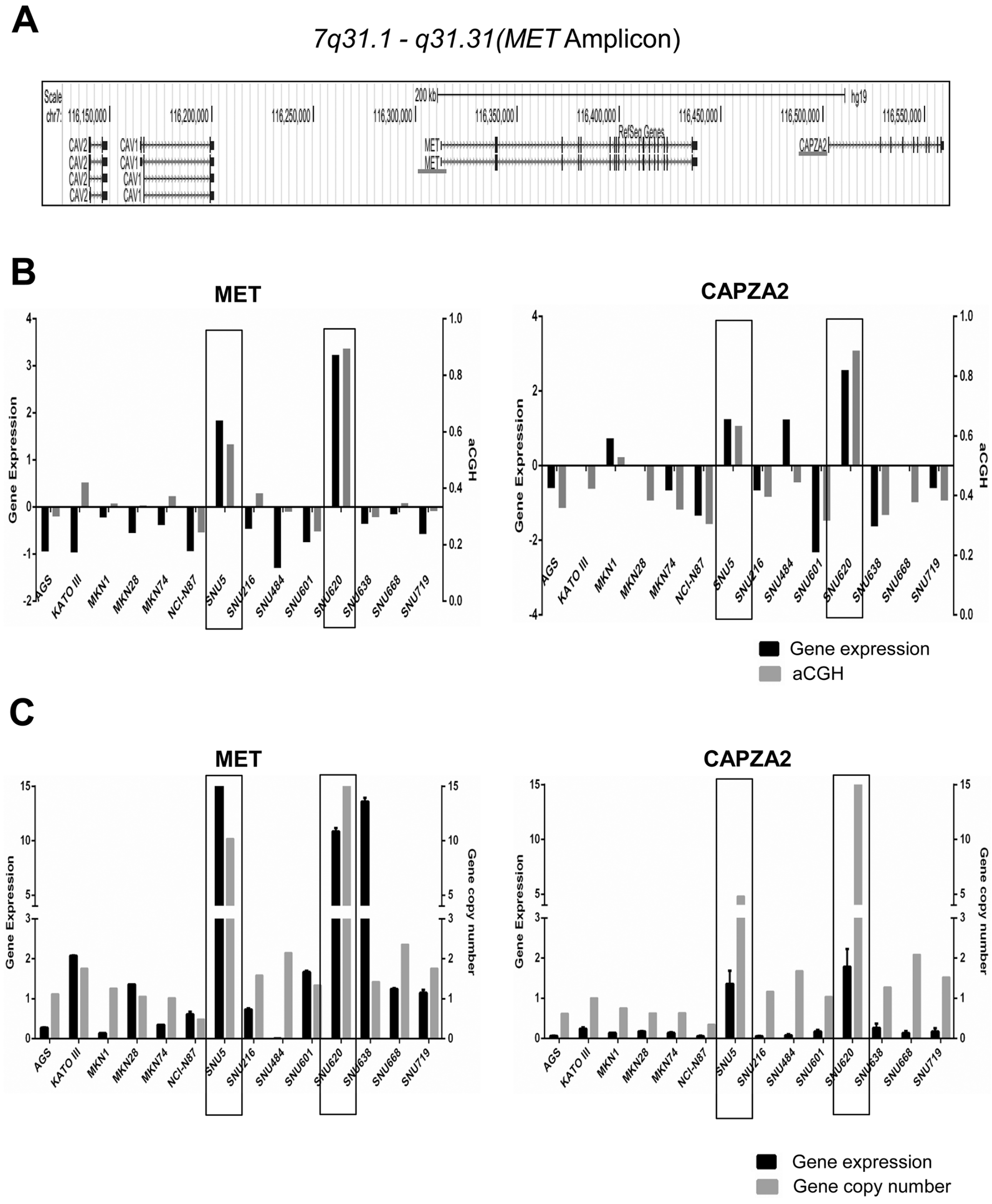

Supplementary Figure 1: Validation of DNA CNAs and gene expression of *MET* and *CAPZA2* in 14 GC cell lines. (A) Snapshot of the *MET* amplicon harboring *MET* and *CAPZA2* in the UCSC genome browser. **(B)** Correlation between DNA CNAs and expression of *MET* and *CAPZA2* across 14 GC cell lines (determined using the HEEBO chip). **(C)** Gene copy number and expression level of *MET* and *CAPZA2* in the 14 GC cell lines, as measured by qPCR and qRT-PCR, respectively.

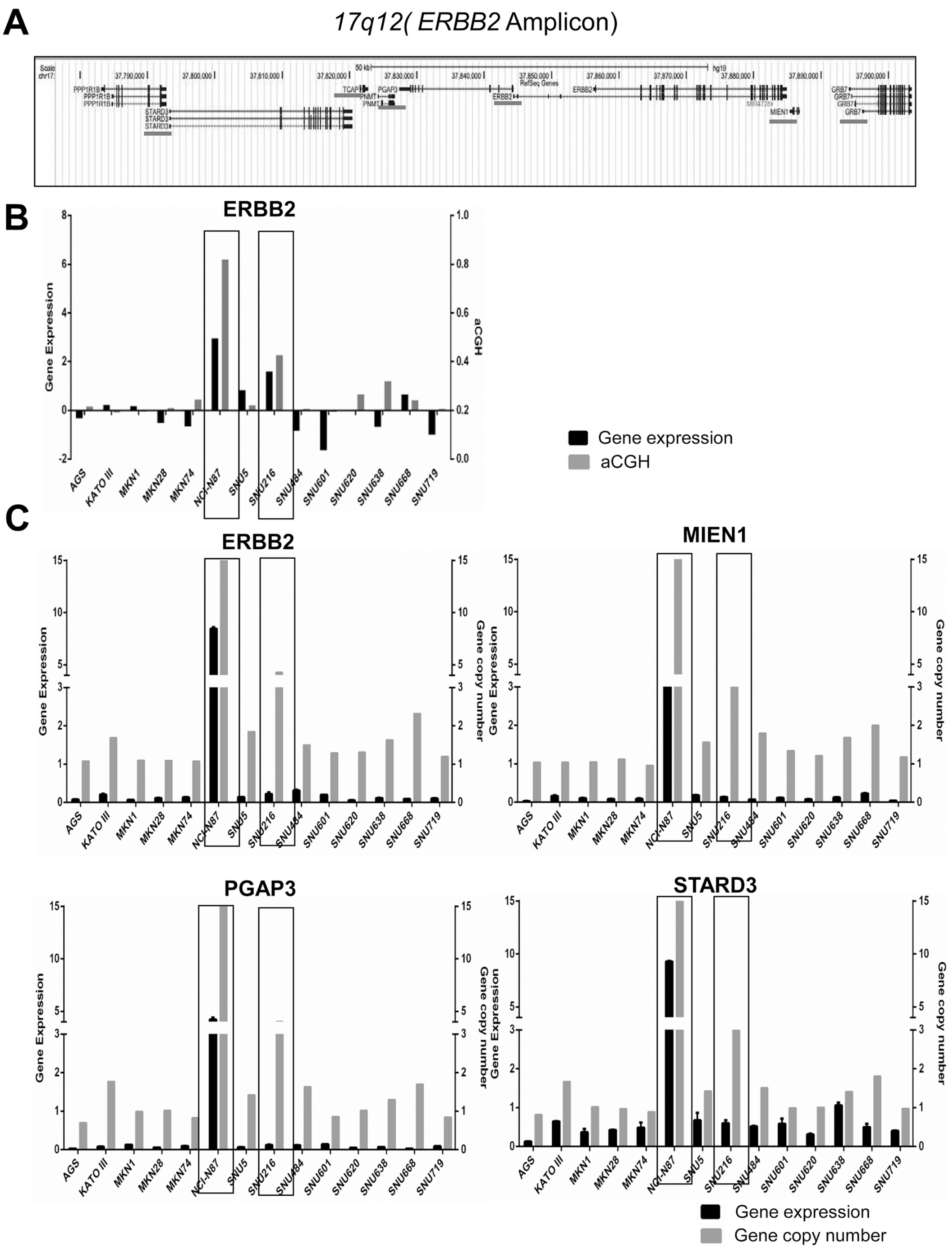

Supplementary Figure 2: Validation of DNA CNAs and gene expression of *ERBB2* and genes co-amplified with *ERBB2* in 14 GC cell lines. **(A)** Snapshot of the *ERBB2* amplicon harboring *ERBB2*, *GRB7*, *MIEN1*, *PGAP3*, and *STARD3* in the UCSC genome browser. **(B)** Correlation between DNA CNA and gene expression of *ERBB2* across 14 GC cell lines (determined using the HEEBO chip). **(C)** Gene copy number and expression levels of *ERBB2*, *MIEN1*, *PGAP3*, and *STARD3* in the 14 GC cell lines, as measured using qPCR and qRT-PCR, respectively.

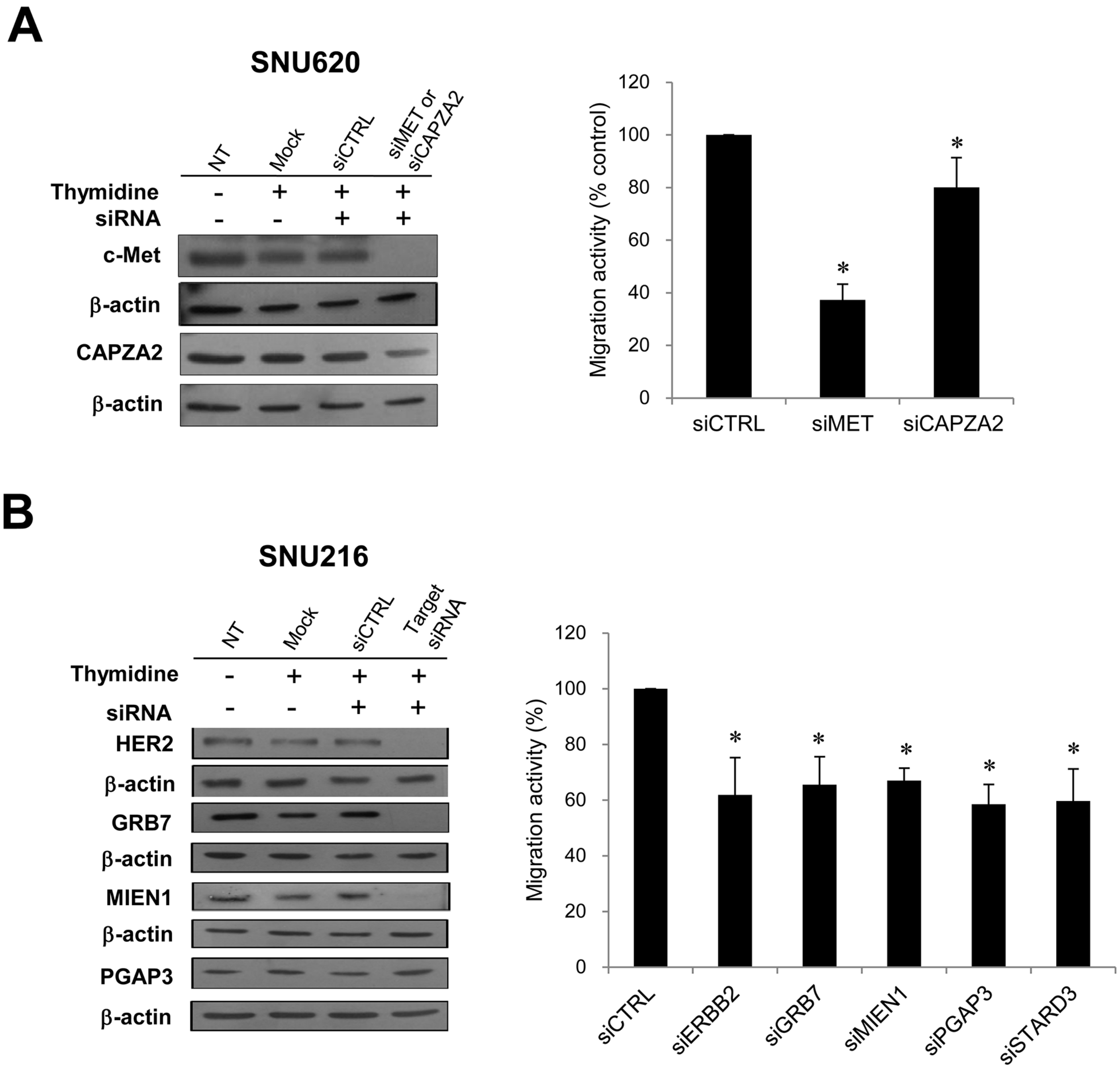

Supplementary Figure 3: Effect of knocking down genes co-amplified with *MET* or *ERBB2* on GC cell migration. **(A)** Effect of *MET* or *CAPZA2* knockdownon migration of SNU620 cells. **(B)** Effect of *ERBB2*, *GRB7*, *MIEN1*, *PGAP3*, or *STARD3* knockdown on migration of SNU216 cells. Depletion or marked reductions in target protein levels in target siRNA-treated cells compared with control siRNA-treated cells were confirmed by western blotting (the exception was STARD3). Significant knockdown of *STARD3* at the mRNA level was confirmed by qRT-PCR (data not shown). Data are presented as the mean ± SD of three or four experiments. Mann-Whitney test, $^{*}P < 0.05$. NT, no treatment.

Supplementary Table 1: Histopathological classification of GC samples included in the analysis.

See Supplementary File 1

Supplementary Table 2: Frequency of DNA CNAs in 38 GC samples.

See Supplementary File 2

Supplementary Table 3: Highly amplified regions and homozygous deleted regions in GC samples.

See Supplementary File 3

Supplementary Table 4: Oncogenes in distinct regions between young and old patients with GC.

See Supplementary File 4

Supplementary Table 5: Correlation between DNA CNAs and gene expression.

See Supplementary File 5

Supplementary Table 6: Primer and probe sequences used for qPCR and qRT-PCR in this study.

See Supplementary File 6
